# sEMG-Based Gesture Recognition Using Sigimg-GADF-MTF and Multi-Stream Convolutional Neural Network

**DOI:** 10.3390/s25113506

**Published:** 2025-06-02

**Authors:** Ming Zhang, Leyi Qu, Weibiao Wu, Gujing Han, Wenqiang Zhu

**Affiliations:** 1 School of Electronic & Electrical Engineering, Wuhan Textile University, Wuhan 430200, China; zhangming@wtu.edu.cn (M.Z.); 2215053010@mail.wtu.edu.cn (L.Q.); wbwu@wtu.edu.cn (W.W.); gjhan@wtu.edu.cn (G.H.); 2 State Key Laboratory of New Textile Materials and Advanced Processing Technologies, Wuhan Textile University, Wuhan 430200, China

**Keywords:** sEMG signal, gesture recognition, multi-stream convolutional neural network (MSCNN), Gramian angular difference field (GADF), Markov transition field (MTF)

## Abstract

To comprehensively leverage the temporal, static, and dynamic information features of multi-channel surface electromyography (sEMG) signals for gesture recognition, considering the sensitive temporal characteristics of sEMG signals to action amplitude and muscle recruitment patterns, an sEMG-based gesture recognition algorithm is innovatively proposed using Sigimg-GADF-MTF and multi-stream convolutional neural network (MSCNN) by introducing the Sigimg, GADF, and MTF data processing methods and combining them with a multi-stream fusion strategy. Firstly, a sliding window is used to rearrange the multi-channel original sEMG signals through channels to generate a two-dimensional image (named Sigimg method). Meanwhile, each channel signal is respectively transformed into two-dimensional subimages using Gram angular difference field (GADF) and Markov transition field (MTF) methods. Then, the GADF and MTF images are obtained using a horizontal stitching method to splice these subimages, respectively. The Sigimg, GADF, and MTF images are used to construct a training and testing dataset, which is then imported into the constructed MSCNN model for experimental testing. The fully connected layer fusion method is utilized for multi-stream feature fusion, and the gesture recognition results are output. Through comparative experiments, an average accuracy of 88.4% is achieved using the Sigimg-GADF-MTF-MSCNN algorithm on the Ninapro DBl dataset, higher than most mainstream models. At the same time, the effectiveness of the proposed algorithm is fully verified through generalization testing of data obtained from the self-developed sEMG signal acquisition platform with an average accuracy of 82.4%.

## 1. Introduction

Surface electromyography (sEMG), as one of the bioelectric signals, exhibits a close relationship with human movements and behaviors. Through the analysis of sEMG signals, researchers can identify human movements and infer behavioral intentions. In recent years, sEMG-based gesture recognition has emerged as a cutting-edge research direction in human–computer interaction technology, finding applications in industrial production, rehabilitation medicine, virtual reality, and other domains. Meanwhile, some artificial intelligence technologies are gradually being applied in human motion classification and analysis [1,2]. Especially as the demand for high recognition accuracy increases in these application scenarios, deep learning networks, particularly convolutional neural networks (CNNs), have attracted significant attention from researchers [3,4].

Currently, CNN-based models have been proposed for recognizing sEMG-based gestures, which can offer notable advantages [5]. For example, the CNN-based models can eliminate the need for manual feature design, automatically screening and extracting multiple features of sEMG signals. These approaches not only avoid tedious feature selection but also ensure the comprehensiveness and completeness of the extracted features. Moreover, the local connectivity, weight sharing, pooling for dimensionality reduction, and multi-layer structure of CNNs enable them to sensitively capture the local characteristics of sEMG signals and detect subtle changes within the signals. Consequently, they can obtain detailed information that is difficult to achieve through manual feature extraction. When extracting features from sEMG signals, CNNs can identify spatial features while assigning equal importance to gesture information regardless of position, thereby enhancing gesture recognition accuracy. Research has shown that deep learning networks exhibit excellent applicability in addressing sEMG-based gesture recognition problems. However, certain limitations remain that require urgent attention [6].

In the process of feature extraction for sEMG signals, features are typically selected from either the time-domain or frequency-domain characteristics of the signals to serve as input for deep learning network models. However, individual time-domain or frequency-domain features fail to fully encapsulate the comprehensive information contained within sEMG signals, leading to potential feature loss. For instance, traditional CNN architectures often neglect time-domain features when extracting features due to their focus on frequency-domain characteristics. Conversely, if only time-domain features are considered, the frequency-domain information of sEMG signals is forfeited. Additionally, research has revealed that sEMG signals are constrained by sampling frequency, which can easily induce frequency aliasing during data preprocessing, thereby hindering the extraction of frequency-domain features. Notably, time-domain features contribute more significantly than frequency-domain features, a conclusion supported by some studies [7].

Both the Gramian angular field (GAF) and Markov transition field (MTF) represent methodologies for time-domain feature extraction, capable of transforming one-dimensional time-series signals into two-dimensional spatial representations. These representations reflect the structural and state transition characteristics of signals over time, enabling the mining of structured signal features while preserving temporal dependencies through the conversion of temporal data into two-dimensional images. To this end, Fu et al. reported the use of a combination of the Gramian angular summation field with linear discriminant analysis (GASF-LDA) to enhance sEMG signal features and improve gesture recognition accuracy [8]. Furthermore, Fan et al. introduced a gesture recognition approach based on GAF-CNN, which utilizes GAF transformation to extract advanced semantic features pertinent to instantaneous values within time-series signals in image form. They incorporated a CNN model for gesture recognition, achieving promising results [9].

To comprehensively harness the temporal, static, and dynamic information features of multi-channel sEMG signals for gesture recognition and acknowledging the sensitivity of sEMG temporal features to action amplitude and muscle recruitment patterns, an sEMG-based gesture recognition algorithm is proposed that integrates Sigimg, GADF, MTF, and multi-stream convolutional neural network (MSCNN). Initially, a sliding window technique is employed to rearrange the multi-channel original sEMG signals through channels to generate a two-dimensional image (named Sigimg method). Simultaneously, GADF and MTF transformations are respectively applied to each channel’s signal, yielding two-dimensional subimages. Then, the subimages are horizontally concatenated to form GADF and MTF images, respectively, which effectively leverage the spatial reorganization of temporal data facilitated by GADF and MTF, thereby enhancing the expressive power of the signal’s temporal features. Based on these three types of images, a training and testing dataset is constructed. On this foundation, the MSCNN model comprises three branch network modules, with multi-stream feature fusion occurring through fully connected layer integration to determine the ultimate recognition outcome. Experimental results on the Ninapro DB1 dataset demonstrate that the proposed algorithm achieves superior recognition accuracy compared to existing algorithms and also exhibits commendable recognition performance on a self-collected dataset.

The sEMG-based gesture recognition algorithm proposed in this paper adopts the Sigimg-GADF-MTF-MSCNN model, which has the following advantages:

(1) In terms of feature extraction from sEMG signals, Sigimg images are employed to characterize sEMG signals, thereby maximizing the preservation of temporal information features inherent in multi-channel sEMG signals;
(2) GADF and MTF images are designed to optimize the extraction of both static and dynamic information features from multi-channel sEMG signals;
(3) In classifier design, the MSCNN, renowned for its exceptional image processing capabilities, is utilized to automatically extract and learn features from Sigimg, GADF, and MTF images. This method circumvents the complexities and limitations associated with manual feature extraction from signals;
(4) The recognition performance is improved by learning and integrating various time-domain features of sEMG signals within the network.

The remainder of this paper is organized as follows: Section 2 reviews the literature pertinent to this study. Section 3 delves into the dataset and primary methodologies employed in this study. Section 4 presents diverse experimental results. Lastly, Section 5 concludes the paper and suggests avenues for further improvement.

## 2. Related Work

The existing sEMG-based gesture recognition algorithms primarily encompass machine learning and deep learning approaches. Traditional machine learning methods heavily rely on manual expertise to extract features in the time-domain, frequency-domain, and time–frequency domain for classification purposes. Common techniques include linear discriminant analysis [10], K-nearest neighbor algorithm [11], support vector machines [12], and random forests [13]. These traditional methods, which depend on human expertise for feature extraction, struggle to adapt to individual patient differences and exhibit limited generalization capabilities. However, deep learning methods do not rely on human expertise for feature extraction and are capable of automatically learning more intricate feature information from data. With the rapid advancement of CNNs, the MSCNN has been continuously proposed and widely adopted. Wei et al. introduced an MSCNN model for the sEMG-based gesture recognition algorithm, which decomposes the original sEMG image into streams of equal size. For each stream, it independently learns representative features through the CNN for each branch. Subsequently, the features learned from all streams are fused into a unified feature map, which is then fed into a fusion network for gesture recognition. Experimental results have demonstrated that the MSCNN outperforms single-stream CNNs (SSCNN) in terms of performance [14]. Wei et al. also reported a novel multi-view CNN model that integrates the classic sEMG feature set with an MSCNN model to fuse the learned multi-view deep features. Experiments showed that the multi-view model outperforms the single-view method in both single-modal and multi-modal sEMG data stream testing [15]. Wei et al. further reported a hierarchical view pooling network for sEMG-based gesture recognition. This network enhances the accuracy of gesture recognition by learning view-specific depth features and view-shared depth features from the hierarchically pooled multi-view feature space [16]. Yang et al. introduced a dynamic sEMG-based gesture recognition algorithm using a multi-stream residual network and short-term memory (MResLSTM), which can extract spatiotemporal features from both global and depth perspectives and combine feature fusion to preserve key information, achieving impressive performance [17]. Wang et al. reported an improved multi-stream convolutional block attention module (MCBAM) for sEMG-based gesture recognition. For multi-channel sEMG and acceleration (ACC) signals, this method embeds the CBAM into the gated recurrent unit (GRU) module to form an MCBAM and GRU (MCBAM-GRU) model, which improves the accuracy of gesture recognition [18]. Jiang et al. presented an sEMG-based gesture recognition algorithm utilizing multi-scale fusion convolution and channel attention, which integrates an efficient channel attention (ECA) module. The model exhibited excellent recognition accuracy and strong generalization ability in experiments [19]. Jiang et al. also reported an sEMG-based gesture recognition algorithm using narrow kernel dual-view feature fusion CNN (NKDFF-CNN). This model employs narrow kernel convolution to learn time-dependent features in each independent channel of sEMG signals, thereby obtaining representative correlation information between specific muscles and gestures. Then, a dual-view structure is utilized to capture shallow and deep features and fuse them at the decision layer. Experimental verification has shown that this method achieves excellent recognition performance in both accuracy and universality [20]. Chen et al. reported the construction of two models using a multi-stream fusion strategy: one is the MultiConvEMG model based on multi-stream deep separable convolution, and the other is the MultiLSTMEMG model based on multi-stream LSTM. Experimental results indicate that, compared to other models, this model offers advantages in accuracy, immediacy, or training cost [21]. Xu et al. reported the use of dual-stream CNN (DSCNN) for feature extraction, fusion, and classification of sEMG and inertial measurement unit (IMU) images, respectively. The experiments demonstrated that the proposed method exhibits superior performance in gesture recognition [22]. Shin et al. reported an sEMG-based gesture recognition system utilizing a four-stream deep learning architecture, where each stream strategically combines time-varying features using temporal convolutional networks (TCNs) with frame-by-frame features using CNNs. The experimental results demonstrated the superiority of the proposed model [23]. Gan et al. introduced an sEMG-based gesture recognition network (SGRN) with multi-dimensional feature extraction and multi-branch information fusion, which integrates the well-constructed spatial feature extraction network (SNet), temporal feature extraction network (TNet) and spatiotemporal feature fusion network (STNet) have demonstrated the effectiveness of SGRN [24].

In summary, the exceptional performance of MSCNN in mining feature information has garnered widespread attention, and the introduction of the MSCNN model for sEMG-based gesture recognition has yielded promising results. It is also evident that existing MSCNN-based gesture recognition algorithms focus more on optimizing the model structure and often employ a single sEMG data processing approach, which may lead the model to overlook the strong temporal correlation between gesture actions and muscle tissues during feature extraction or to lose shallow feature information as the network layers deepen, ultimately limiting recognition accuracy.

## 3. Methods

### 3.1. Experimental Data

Experiments were conducted using multi-channel sEMG signals from the publicly available Ninapro DB1 dataset. This dataset comprises data from 27 healthy subjects (20 males, 7 females; 25 right-handed, 2 left-handed, with an average age of 28 ± 3.4 years) who performed 52 finger, hand, and wrist movements. The data were acquired using a 10-channel Otto Bock electrode with a sampling rate of 100 Hz. The movements were categorized into three types of exercises, including basic finger movements, grasping movements, and functional gestures. Each type of movement was repeated 10 times, lasting 5 s per repetition with a rest interval of 3 s. The original sEMG signals underwent amplification, bandpass filtering, and root mean square (RMS) correction. Detailed definitions and images of the gesture movements are provided by Atzori et al. [25]. Due to the complexity of utilizing the original signal format as direct input for the model, data segmentation was performed in advance. In this study, data segmentation was conducted based on subject, stimulus (the original label of the movement), and repetition for subsequent data processing. The final data were stored in .mat files, with each file corresponding to a specific combination of subject, stimuli, and repetition (e.g., 001_002_003.mat represents the third repetition of the second stimulus for the first subject).

### 3.2. Multi-Channel Data Processing

#### 3.2.1. Conversion of Multi-Channel Signals to Sigimg Images

**Figure 1:**
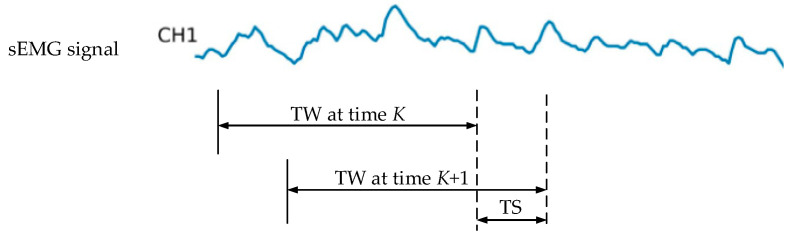
sEMG signal segmentation using the sliding window method. Capture the sEMG signal according to the length of the time window (TW) and slide the time window in the time stride (TS).

For both single-channel and multi-channel sEMG signals, a sliding window method was employed for windowed sampling. Englehart et al. highlighted in their early research on sEMG-based control systems that the control delay should not exceed 300 ms. To meet this constraint, a sliding window with a length of 200 ms was primarily utilized with a stride of 100 ms [26]. This process is illustrated in Figure 1, where TW (time window) and TS (time stride) denote the length of the time window and the incremental step at time point K, respectively.

**Figure 2:**
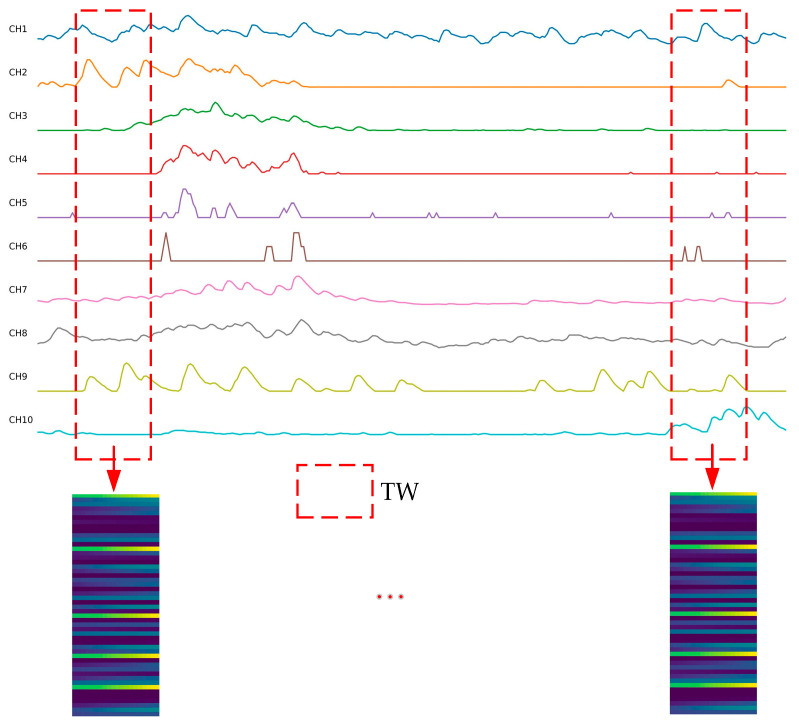
Flowchart to generate a Sigimg image for multi-channel sEMG signals. Rearrange the multi-channel sEMG signals of the time window length by channels to generate a Sigimg image.

To enable CNNs to better capture the correlations between sEMG signals collected from different electrodes, the algorithm presented by Jiang et al. [27] was adopted to transform multi-channel one-dimensional signals within a time window into two-dimensional images. This process is depicted in Figure 2. Specifically, the original multi-channel sEMG signals were rearranged by channel to generate a Sigimg image. In this image, each signal sequence has the opportunity to be adjacent to other sequences, facilitating CNNs in extracting hidden correlations between adjacent signals. For the 10-channel sEMG signals from the Ninapro DB1 dataset, the window length was set to 200 ms (i.e., 20 sampling points), and the window size 20×10 was determined accordingly. The Sigimg image in a time window represents a specialized arrangement of sEMG signal channels with the size of 20×100.

#### 3.2.2. Conversion of Multi-Channel Signals to GADF and MTF Images

The GAF method can effectively display the fluctuations in one-dimensional signals in the GAF image and record sEMG signal information. Scale the values $x_{i}$ (i=1,⋯,n) in the one-dimensional data sequence $X=\left\{x_{1},x_{2},\cdots ,x_{n}\right\}$ to between [−1, 1], which is calculated using Equation (1).

(1) $${\tilde{x}}_{i}=\frac{x_{i}-\max X+x_{i}-\min X}{\max X-\min X}$$

**Figure 3:**
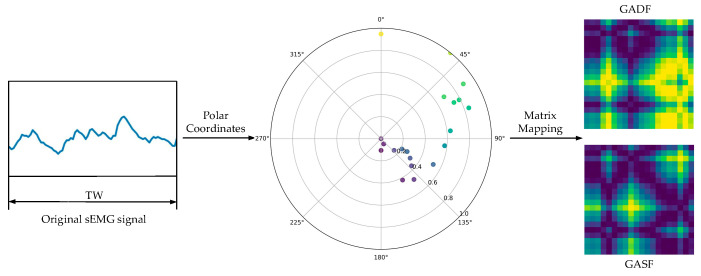
GASF/GADF transform.

The value in the scaled data sequence is used as the cosine value of the angle ϕ, and when it is scaled to [−1,1], the angle range is [0,π]. Set the timestamp $t_{i}$ to the polar coordinate radius r and convert the one-dimensional signal into a polar coordinate system, as shown in Equation (2).

(2) $$\left\{\begin{array}{l} \phi =\arccos{\tilde{x}}_{i};-1\leq {\tilde{x}}_{i}\leq 1,{\tilde{x}}_{i}\in \tilde{X}\\ r=\frac{t_{i}}{N};t_{i}\in N \end{array}\right.$$

where N is the constant factor of the normalized polar coordinate generation space, and $\tilde{X}$ is the normalized scaled X. In this context, GAF is divided into two forms: GASF and GADF. Although their conversion principles and algorithms are similar, as shown in Figure 3. GADF more effectively captures the relative changes between adjacent samples within the same time period of the signal. Consequently, GADF images contain more detailed feature information compared to GASF images. After encoding, the temporal correlation from the top-left corner to the bottom-right corner of GADF images is preserved, and the main diagonal lines retain the original information of the time-series signals [28]. Therefore, GADF was used for calculation and polar coordinate transformation in this study. The definition of the Gram matrix is presented in Equation (3).

(3) $$G=\left[\begin{array}{ccc} \cos(\phi_{1}-\phi_{1}) & \vdots & \cos(\phi_{1}-\phi_{n})\\ \cos(\phi_{2}-\phi_{1}) & \vdots & \cos(\phi_{2}-\phi_{n})\\ \vdots & \cos(\phi_{i}-\phi_{i}) & \vdots \\ \cos(\phi_{n}-\phi_{1}) & \vdots & \cos(\phi_{n}-\phi_{n}) \end{array}\right]$$

Markov transition field (MTF) is an image encoding method for time-series signals based on the Markov transition matrix, which regards the time-series signals as a Markov process. Under the condition of knowing the current state, its future evolution does not depend on its previous evolution. Therefore, a Markov transition matrix is constructed and then extended to the Markov transition field to achieve image coding [29]. For the one-dimensional data sequence $X=\left\{x_{1},x_{2},\cdots ,x_{n}\right\}$, it is divided into Q quantile units according to the numerical range, and each value is quantified by the quantile $q_{j}$ (j∈1,⋯,Q). Then, the value $x_{i}$ (i=1,⋯,n) in the X corresponds to a unique quantile $q_{i}$. Thus, a Markov transition matrix $W_{QQ}$ from $w_{ij}$ with a size of Q×Q is built, where $w_{ij}$ is determined by the probability P of the value $x_{i}$ corresponding to $q_{i}$ being followed by $q_{j}$. That is, $w_{ij}$ in the MTF denotes the transition probability of $q_{i}\to q_{j}$, as shown in Equation (4).

(4) $$w_{ij}=P(x_{i}\in q_{i}\left|x_{i-1}\in q_{j}\right.)\;s.t.\;\sum_{j}w_{ij}=1$$

A Markov transition field matrix with a size of n×n is defined by arranging each $w_{ij}$ in chronological order, as shown in Equation (5).

(5) $$M=\left[\begin{array}{ccc} w_{ij}\left|x_{1}\in q_{i},\right.x_{1}\in q_{j} & \vdots & w_{ij}\left|x_{1}\in q_{i},\right.x_{n}\in q_{j}\\ w_{ij}\left|x_{2}\in q_{i},\right.x_{1}\in q_{j} & \vdots & w_{ij}\left|x_{2}\in q_{i},\right.x_{n}\in q_{j}\\ \vdots & \ddots & \vdots \\ w_{ij}\left|x_{n}\in q_{i},\right.x_{1}\in q_{j} & \vdots & w_{ij}\left|x_{n}\in q_{i},\right.x_{n}\in q_{j} \end{array}\right]$$

**Figure 4:**
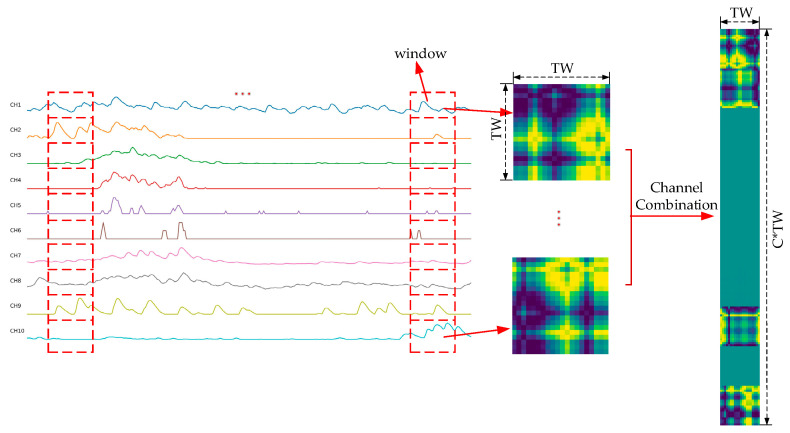
Flowchart to generate a GADF image for multi-channel sEMG signals. Firstly, convert the single-channel sEMG signals into GADF subimages according to the length of the time window, and then stack these GADF subimages into a GADF image.

**Figure 5:**
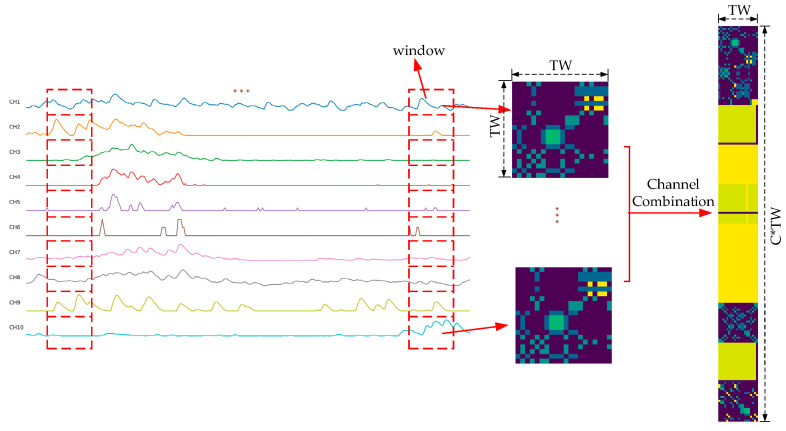
Flowchart to generate an MTF image for multi-channel sEMG signals. Firstly, convert the single-channel sEMG signals into MTF subimages according to the length of the time window, and then stack these MTF subimages into an MTF image.

The process of converting the original multi-channel sEMG signals into GADF and MTF images is shown in Figure 4 and Figure 5. For the one-dimensional sEMG signal with a window length of n, a matrix of [n,n] is generated through GADF transformation to obtain a n×n-sized image. For the processing of multi-channel sEMG signals, each channel is separately converted into a subimage and then horizontally concatenated to a GADF image. Therefore, for C channels, the GADF transformation should be applied individually to each channel. Subsequently, the resulting outputs can be integrated to form a single GADF image with a dimension of n×(C*n). For the 10-channel sEMG signals from the Ninapro DB1 dataset, 20 data points are firstly extracted with a window length in channel order. Subsequently, a GADF subimage with a size of 20×20 is generated by performing GADF transformation. The generated 10 subimages are horizontally stitched together to form a GADF image with a size of 20×200. The generation process of MTF images is the same as that of GADF images and will not be repeated here.

#### 3.2.3. Dataset Construction

After obtaining the original multi-channel sEMG signals, Sigimg, GADF, and MTF images are obtained within each sliding window using the above processing methods. At the same time, there are three types of image encoding, so more feature information can be obtained during the convolution process. The adjusted images are normalized to construct a dataset that can be imported into CNNs for training and testing.

### 3.3. Structure Design of CNNs

#### 3.3.1. Multi-Stream CNN

**Figure 6:**
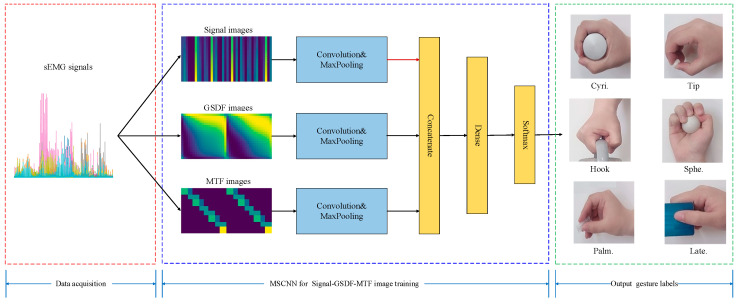
sEMG-based gesture recognition framework using the Sigimg-GADF-MTF-MSCNN algorithm.

Taking inspiration from the excellent performance of CNNs in sEMG-based gesture recognition, we constructed a gesture recognition framework using the Sigimg-GADF-MTF-MSCNN algorithm, as illustrated in Figure 6. The Sigimg, GADF, and MTF images were employed as inputs for feature extraction and network learning across the three branches of the MSCNN model. The features of the Sigimg, GADF, and MTF images learned by the network were merged into a fully connected layer for fusion and finally output results of gesture recognition. The MSCNN architecture primarily comprises convolutional layers, pooling layers, and fully connected layers. In the convolutional layers, the convolution kernels perform operations to extract features from input samples. The pooling layers utilize spatial pooling rules to reduce the dimensions of the feature maps generated by the convolutional layers. Following convolution and pooling, fully connected layers are used to aggregate the feature space information derived from the extracted features.

**Figure 7:**
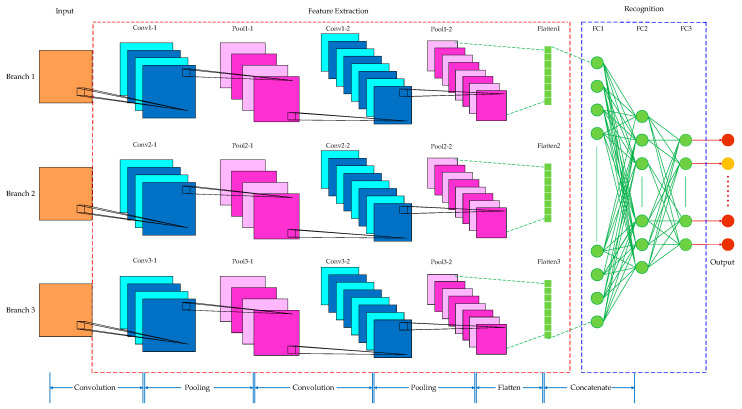
Main structure of the proposed MSCNN model.

As shown in Figure 7, the MSCNN model constructed in this paper has two convolutional layers and two pooling layers arranged alternately in each branch of the CNNs. The input size of the Sigimg image branch is 20×100×1. For the GADF and MTF image branches, the input size is 20×200×1. In all branches, the first layer uses 16 convolution kernels with a kernel size of 3×3. The subsequent pooling layer performs a maximum-pooling operation with a kernel size of 2×2. The second layer in each branch utilizes 32 convolution kernels, where the input size for the Sigimg image branch is 3×3, and for the GADF and MTF image branches, it is 5×5. The subsequent pooling layer in each branch applies a maximum-pooling operation with the same kernel size of 2×2. By doing so, the model can obtain more temporal feature information from the Sigimg image and more static and dynamic feature information from the GADF and MTF images, respectively.

After flattening the image features output by the three CNN branches, they are transformed into feature vectors. Subsequently, a fully connected layer concatenates, fuses, and recognizes these feature vectors. Following three fully connected operations, the corresponding values of 52 categories are output to achieve the final gesture recognition result.

The layers and parameters of the MSCNN model trained on the Ninapro DB1 dataset are summarized in Table 1, where *s* represents the stride of the convolution, *p* represents the padding of the convolution, and *c* represents the number of output channels of the convolution. A rectified linear unit (ReLU) serves as the non-linear activation function. Additionally, batch normalization (BN) and random dropout operations are integrated into the MSCNN model to enhance the convergence speed and generalization capability of the network model training.

#### 3.3.2. Hyperparameter Setting of the Network

When the partial parameters (such as network layers) are fixed, the optimization algorithm with adaptive moment estimation (Adam) is adopted, and the cross entropy loss function is selected as the loss function for network training [30]. For the learning rate, a segmented decay strategy is adopted with an initial value of 0.001–0.01 and a decay of 0.1 every 5–10 epochs. For batch sizes, 32–128 is preferred to balance training speed and gradient stability. For the dropout rate, 0.3–0.5 is set before the fully connected layer to avoid feature redundancy. Finally, the batch size is set to 100, and the number of iterations is set to 30. To accelerate convergence, a learning rate decay strategy was adopted, where the initial learning rate was set to 0.1, and the learning rate was divided by 10 at the 16th and 24th iterations, respectively. For all neural network hidden layers that use random deactivation regularization, the proportion of randomly deactivated neurons is 50%.

## 4. Experiments and Analysis

### 4.1. Evaluation Indicators

All experiments were conducted on a workstation equipped with an Intel^®^ Core ^TM^ (Intel, Santa Clara, CA, USA) i7-8650U CPU, 24GB of memory, and a single RTX 3060Ti GPU with 12GB of memory. The operating system of the workstation was Windows 10. The software environment was Python 3.8.5, and the MxNet 1.6.0 framework was used for model construction.

In the experiments, gesture recognition accuracy was adopted as the primary metric to evaluate the proposed algorithm. For the *i*-th subject, the formula for calculating gesture recognition accuracy $A_{i}$ is calculated using Equation (6).

(6) $$A_{i}=\frac{S_{i}}{\sum_{i=1}^{m}S_{i}}$$

where $S_{i}$ represents the number of correctly identified samples for the *i*-th subject. And m denotes the total number of samples for the *i*-th subject. The gesture recognition accuracy was obtained by conducting gesture recognition tests on each subject in the Ninapro DB1 dataset. The final accuracy $\bar{A}$ is computed as the average of the gesture recognition accuracies $\left\{A_{1},A_{2},\cdots ,A_{n}\right\}$ across all subjects, as shown in Equation (7).

(7) $$\bar{A}=\frac{\sum_{i=1}^{n}A_{i}}{n}$$

where n is the total number of subjects.

To further compare the performance between different algorithms, Precision and Recall metrics are used for evaluation, as defined in Equations (8) and (9). In addition, the indicators such as MACs (multiply–accumulate operations), training time, testing time, and inference time are also considered. MACs represent the computational complexity of a model, in which one MAC consists of two basic operations: one multiply and one accumulate, measured in millions (M). The training time represents the average time required for all subjects to train in seconds (s). The testing time represents the average time required for all subjects to train in seconds (s). The inference time represents the duration required for a single sample of the input model and outputs the results in milliseconds (ms).

(8) $$P_{\mathrm{recision}}=\frac{TP}{TP+FP}$$

(9) $$R_{\mathrm{ecall}}=\frac{TP}{TP+FN}$$

where *TP* (true positive) is the number of positive cases recognized as positive; *FP* (false positive) is the number of negative cases recognized as positive; *TN* (true negative) is the number of negative cases recognized as negative; *FN* (false negative) is the number of positive cases recognized as negative.

### 4.2. Network Model Training and Testing

The performance of sEMG-based gesture recognition may be affected by the distribution difference between the training data and testing data. Specifically, for experiments on the NinaPro DB1 dataset, the trials from the 1st, 3rd to 6th, and 8th to 10th repetitions of all 27 subjects are used as the training set, and the trials from the 2nd and 7th repetitions are used as the testing set. When training a deep neural network model, the NinaPro DB1 dataset has been partitioned into a training set and testing set at a ratio of 4:1. Subsequently, the MSCNN model is pretrained using the training dataset to acquire optimized model parameters. During the testing phase of the deep neural network models, the test dataset is compiled by combining the test set samples from all subjects. The MSCNN model is initialized with the pretrained model parameters, and gesture recognition accuracy is ultimately evaluated by conducting tests for each subject.

### 4.3. Experimental Results

#### 4.3.1. Comparative Experiment Between MSCNN, SSCNN, and DSCNN

Firstly, the accuracy of sEMG-based gesture recognition using the proposed MSCNN model is compared with that of the SSCNN and DSCNN models constructed on the basis of each branch structure. This comparison aims to validate the effectiveness of the multi-stream architecture of the MSCNN model. The specific descriptions of the SSCNN and DSCNN structures used for comparison are as follows:

(1) SSCNN model: Based on the SSCNN model, three gesture recognition algorithms were developed: Sigimg-SSCNN, GADF-SSCNN, and MTF-SSCNN. For the Sigimg-SSCNN algorithm, the input is Sigimg images, and its network architecture consists of two alternating convolutional and pooling layers, followed by one flattening layer and three fully connected layers. The number of neurons in each connected layer is 1024, 256, and 52, respectively. The parameters of each convolution layer and pooling layer in the model are the same as those of Branch1 in Table 1. For the GADF-SSCNN and MTF-SSCNN algorithms, the inputs are GADF and MTF images, respectively. Their network architectures are identical, comprising two alternating convolutional and pooling layers, one flattening layer, and three fully connected layers. The number of neurons in each connected layer layer is 2048, 512, and 52, respectively. The parameters of each convolution layer and pooling layer in the model are the same as those of Branch2 or Branch3 in Table 1;
(2) DSCNN model: Based on the DSCNN model, two gesture recognition algorithms were constructed: Sigimg-GADF-DSCNN and GADF-MTF-DSCNN. For the Sigimg-GADF-DSCNN algorithm, the inputs include Sigimg and GADF images, and its network architecture consists of two branches. The parameters of its convolutional, pooling, and flattening layers are identical to those of Branch1 and Branch2 in Table 1. The number of neurons in the three fully connected layers is 4096, 1024, and 52, respectively. Similarly, the GADF-MTF-DSCNN algorithm uses GADF and MTF images as inputs and has a two-branch architecture. Its convolutional, pooling, and flattening layer parameters are consistent with those of Branch2 and Branch3 in Table 1. The number of neurons in the three fully connected layers is also 4096, 1024, and 52, respectively.

**Figure 8:**
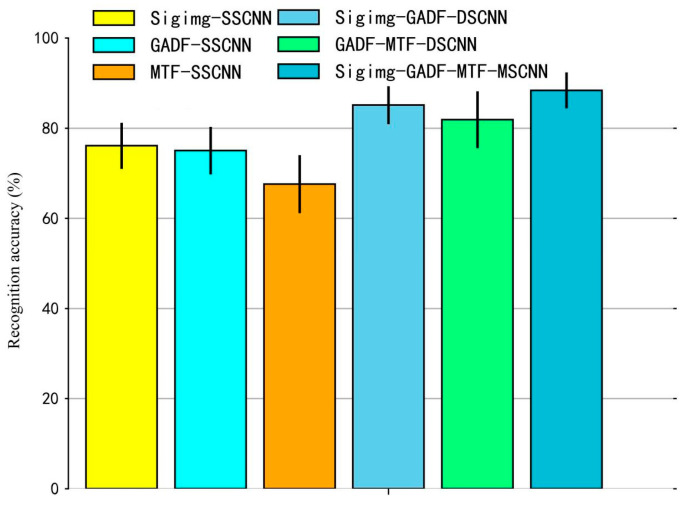
Comparison results of gesture recognition accuracy of various algorithms on the Ninapro DB1 dataset. The height of each column represents the average accuracy, while the error bar represents the standard deviation.

The accuracy of sEMG-based gesture recognition using the Sigimg-GADF-MTF-MSCNN algorithm, as well as the Sigimg-SSCNN, GADF-SSCNN, MTF-SSCNN, Sigimg-GADF-DSCNN, and GADF-MTF-DSCNN algorithms, is illustrated in Figure 8 on the Ninapro DB1 dataset. As shown in Figure 8, under identical input conditions of Sigimg, GADF, and MTF images, the recognition accuracy of the Sigimg-GADF-MTF-MSCNN algorithm reaches 88.40±2.01% with a confidence interval (95%) of [86.39% 90.01%], where its average accuracy is higher than the other five algorithms with 3.28% to 20.81%. This demonstrates that the MSCNN model proposed in this study exhibits superior sEMG gesture recognition performance compared to SSCNN and DSCNN models. The experimental results indicate that multi-stream networks can compensate for information deficiencies in single-input data and preserve richer features, thereby enhancing overall recognition accuracy.

#### 4.3.2. Ablation Study

To better validate the effectiveness of the Sigimg-GADF-MTF-MSCNN model for sEMG-based gesture recognition, the following ablation experiments were performed for the six experiments. The sEMG images (Sigimg, GADF, and MTF images) were used as the input source of the model.

Experiment I: Sigimg-SSCNN.
Experiment II: GADF-SSCNN.
Experiment III: MTF-SSCNN.
Experiment IV: Sigimg-GADF-DSCNN.
Experiment V: GADF-MTF-DSCNN.
Experiment VI: Sigimg-GADF-MTF-MSCNN.

The experimental results in Table 2 show that the Precision and Recall metrics using the Sigimg GADF-MTF-MSCNN algorithm reach 88.6% and 78.5%, respectively, which are superior to other single-stream and dual-stream CNN algorithms. This indicates that compared with the single-stream and dual-stream CNN algorithms., the Sigimg-GADF-MTF-MSCNN algorithm significantly improves recognition accuracy. In addition, although the MACs, training time, testing time, and inference time gradually increase with the number of model channels, the inference time required of the input model for the three types of Sigimg, GADF, and MTF images within a time window is 62 ms, which is less than 200 ms, indicating that real-time applications can be met under certain conditions.

#### 4.3.3. Comparative Experiment Between MSCNN and Other Network Models

To further demonstrate the advantages of the proposed model, it is necessary to increase the comparison with other network models, so we conduct experiments on the Ninapro DB1 dataset. The experimental results are presented in Table 2. By analyzing the comparative results, it is evident that the proposed MSCNN model exhibits a slight superiority over other algorithms. As shown in Table 3, Wei’s two proposed models achieved gesture recognition accuracies of 85.0% and 88.2%, respectively, while the MSCNN model proposed in this study achieved an accuracy of 88.4%, which is relatively comparable to Wei’s results. These MSCNN models consist of two primary stages: a multi-stream decomposition stage and a fusion stage. In the decomposition stage, each stream independently learns representative features through the CNN for each branch. Subsequently, in the fusion stage, the learned features from all streams are merged into a unified feature map, which is then fed into the fusion network for gesture recognition. The proposed MSCNN model achieves higher gesture recognition accuracy by effectively mining the temporal, static, and dynamic feature information embedded in multi-channel sEMG signals.

### 4.4. Practical Application Testing

**Figure 9:**
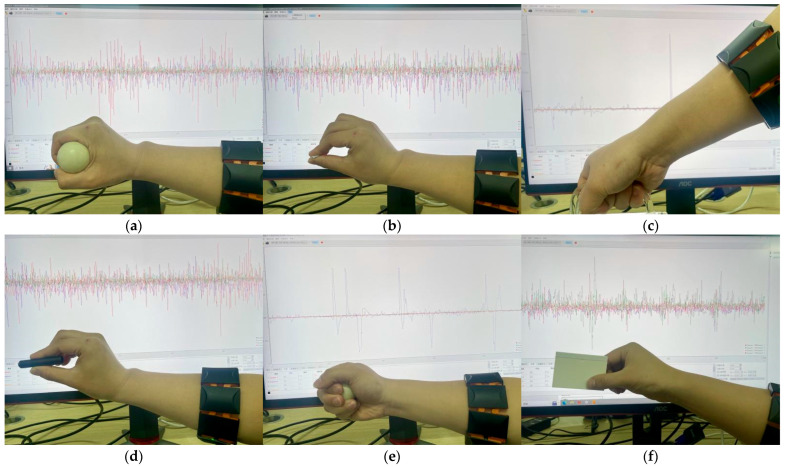
sEMG data collection of six movements: (**a**) Cyri.; (**b**) Tip.; (**c**) Hook; (**d**) Sphe.; (**e**) Palm.; and (**f**) Late.

In order to verify the practical applicability of the algorithm proposed in this paper, an 8-channel EMGRPO arm ring was employed to collect sEMG signals. The system collects sEMG signals from eight channels, with an electrode amplification factor of 2000, a sampling accuracy of 12 bits, and a sampling frequency of 500 Hz. Then, the data are sent to the upper computer serial port through the 5.0 Bluetooth protocol. The hardware collected data are received by the upper computer. Finally, the upper computer saves the received original data in CSV format for subsequent processing. In order to collect data, five healthy participants (two males and three females) of similar age (23 to 25 years old) were asked to repeat the six gesture movements shown in Figure 9: (1) grasping cylindrical objects (Cyril.); (2) holding small objects with fingertips (Tip); (3) hooking heavy objects (Hook); (4) holding small objects facing the palm (Sphe.); (5) grasping spherical objects (Palm.); and (6) grasping thin and flat objects (Late.). This experiment was approved by the Ethics Committee of Wuhan Textile University and the subjects themselves.

**Figure 10:**
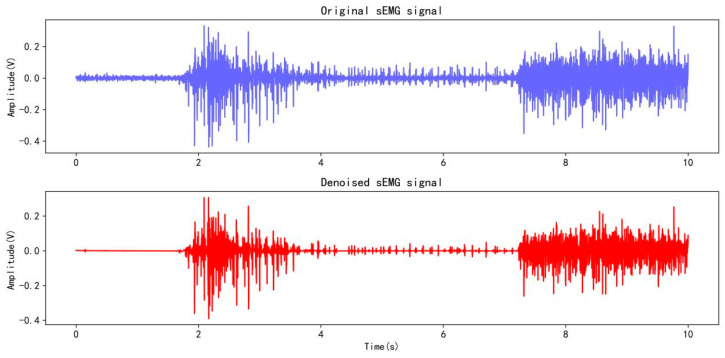
Wavelet denoising on the sEMG signal from a channel.

Following the same experimental collection protocol as Ninapro DB1, subjects repeated each type of action 30 times, with each movement lasting 5 s and a 3 s break interval. For the collected data, the “db4” wavelet is first used for denoising, as shown in Figure 10, and then downsampled to 100 Hz. The data are segmented according to subjects, stimuli, and repetitions for subsequent data processing, and the data are converted into a mat file format for storage. The subjects numbered 1, 3, 4, and 5 of all five subjects were used for training, and subject number 2 was used for testing, which is divided into a training set and a testing set at a ratio of 4:1.

**Figure 11:**
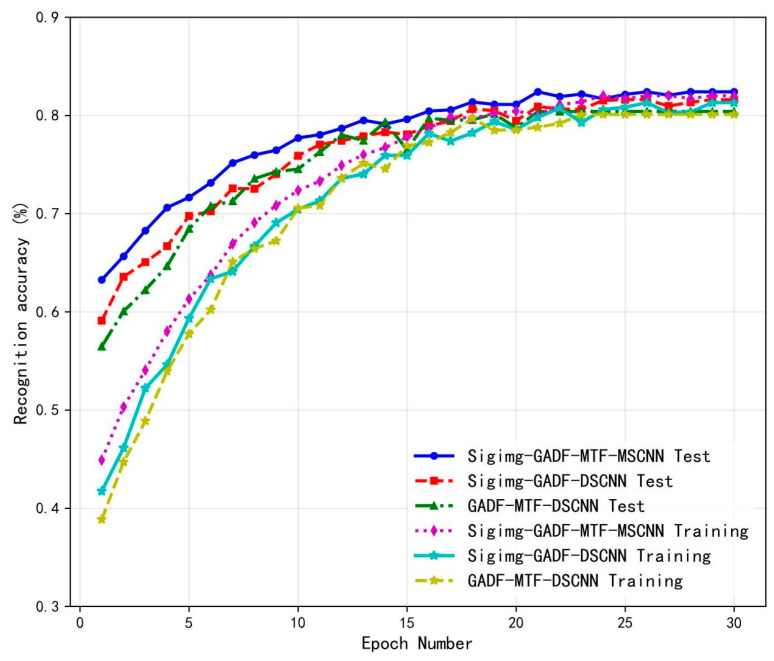
Change curve of recognition accuracy using self-collected data.

To further demonstrate the performance of the proposed Sigimg-GADF-MTF-MSCNN algorithm, a comparative test of recognition performance was conducted against two gesture recognition algorithms: Sigimg-GADF-DSCNN and GADF-MTF-DSCNN. The first test is to import the processed Sigimg, GADF, and MTF images into three models and conduct 30 rounds of iterative training tests. The experimental results show the training test curve in Figure 11. As the number of iterations increases, the fluctuation of the training test curve decreases, and the stability increases. From Figure 11, it can be seen that the Sigimg-GAF-MTF-MSCNN algorithm has a recognition accuracy of 82.4% after 30 rounds of iterative testing, which is higher than the other two algorithms.

**Figure 12:**
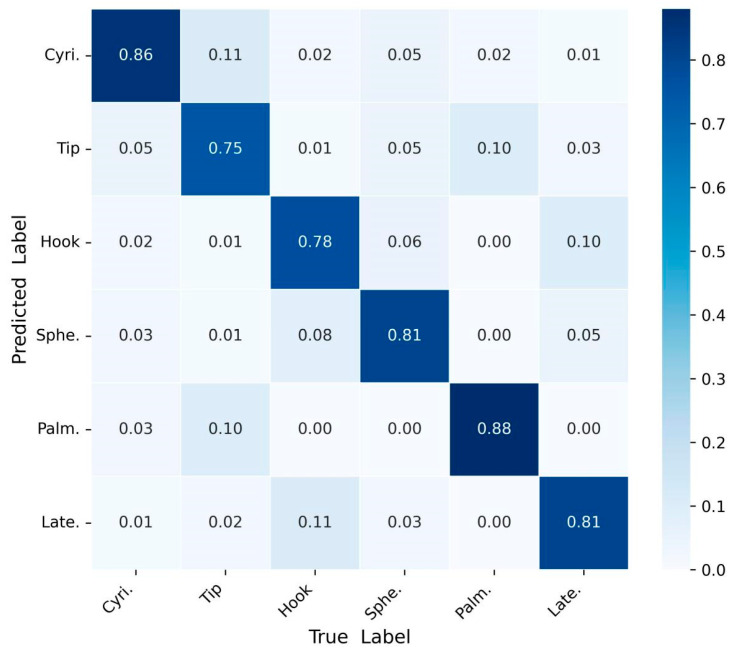
Test results by the confusion matrix method using self-collected data.

The second test is through the five-fold cross-validation method, taking an average classification accuracy of 10 times. Figure 12 shows the confusion matrix of the recognition results of six gesture movements using the Sigimg-GADF-MTF-MSCNN algorithm. Obviously, most of the recognition errors are that the hooking movement is mistaken for the grasping movement, and the palm movement is mistaken for the tip movement [33]. From Figure 12, it can be seen that the Sigimg-GAF-MTF-MSCNN algorithm can recognize six gesture movements, and the recognition accuracy is between 75% and 88%, which shows the feasibility and generalization of the proposed algorithm.

## 5. Conclusions

In this paper, a novel method for sEMG-based gesture recognition using the Sigimg-GADF-MTF-MSCNN algorithm is proposed. The original multi-channel sEMG signals are rearranged to construct the Sigimg, GADF, and MTF images, which are obtained through horizontal concatenation. These images are then fed into the MSCNN model, where the temporal, static, and dynamic information features of sEMG signals are fused via fully connected layers to achieve accurate gesture recognition. Experimental results on the Ninapro DB1 dataset demonstrate the effectiveness of the MSCNN model and its superior performance in gesture recognition tasks. Compared with the SSCNN and DSCNN models, the proposed MSCNN model achieves higher accuracy. Furthermore, tests conducted on self-collected data confirm that the Sigimg-GADF-MTF-MSCNN algorithm satisfies the requirements for recognition accuracy and generalization. It can provide a promising approach for multi-channel sEMG signal processing and gesture recognition.

In practical application scenarios, situations may arise where the training dataset and test dataset originate from different subjects or distinct data acquisition systems. Therefore, further testing and optimization of the proposed gesture recognition algorithm are necessary to enhance its generalization capability and ensure its applicability in the real application environment.

## Figures and Tables

**Table 1:** The layers and parameters of the MSCNN model trained on the Ninapro DB1 dataset.

Branch	Layer Type	Key Parameters	Output Dimension
Branch 1	Conv1-1	Conv 16@3 × 3, s = 1, *p* = 1,c = 16, ReLU	20 × 100 × 16
	Pool1-1	2 × 2 MaxPooling, s = 2	10 × 50 × 16
	Conv1-2	Conv 32@3 × 3, s = 1, *p* = 1,c = 32, ReLU	10 × 50 × 32
	Pool1-2	2 × 2 MaxPooling, s = 2	5 × 25 × 32
	Flatten1	-	4000
Branch 2	Conv2-1	Conv 16@3 × 3, s = 1, *p* = 1,c = 16, ReLU	20 × 200 × 16
	Pool2-1	2 × 2 MaxPooling, s = 2	10 × 100 × 16
	Conv2-2	Conv 32@5 × 5, s = 1, *p* = 2,c = 32, ReLU	10 × 100 × 32
	Pool2-2	2 × 2 MaxPooling, s = 2	5 × 50 × 32
	Flatten2	-	8000
Branch 3	Conv3-1	Conv 16@3 × 3, s = 1, *p* = 1, c = 16, ReLU	20 × 200 × 16
	Pool3-1	2 × 2 MaxPooling, s = 2	10 × 100 × 16
	Conv3-2	Conv 32@5 × 5, s = 1, *p* = 2,c = 32, ReLU	10 × 100 × 32
	Pool3-2	2 × 2 MaxPooling, s = 2	5 × 50 × 32
	Flatten3	-	8000
Concatenate	-	-	20,000
Dense 1	FC1	ReLU	4096
Dense 2	FC2	ReLU	2048
Dense	FC3	Softmax	52

**Table 2:** Comparison results from different algorithms.

Algorithms	$P_{\mathrm{recision}}$	$R_{\mathrm{ecall}}$	MACs (M)	Training Time (s)	Testing Time (s)	Inference Time (ms)
Sigimg-SSCNN	77.9%	67.4%	25.0	6853	1713	19
GADF-SSCNN	75.3%	66.4%	58.2	10,414	2603	28
MTF-SSCNN	63.0%	59.9%	58.2	10,664	2666	29
Sigimg-GADF-DSCNN	85.9%	74.7%	83.2	13,234	3308	36
GADF-MTF-DSCNN	80.1%	71.5%	116.4	15,098	3774	41
Sigimg-GADF-MTF-MSCNN	88.6%	78.5%	141.4	22,943	5736	62

**Table 3:** Comparison results of the different algorithms on the Ninapro DB1 dataset.

Algorithms	Recognition Accuracy (%)
Atzori_Net [5]	66.7
Geng_Net [31]	77.8
Cheng_CNN [32]	82.5
Wei_MSCNN [14]	85.0
Wei_MVCNN [15]	88.2
SGM_MSCNN (Ours)	88.4

## Data Availability

The self-collected data presented in this study are available upon reasonable request from the first author. Currently, some data are available on the website: https://gitee.com/zhang_ming_wtu/s-emg_-gesture_-recognition.git (18 April 2025).

## References

[1] Latreche A., Kelaiaia R., Chemori A., Kerboua A. (2023). Reliability and validity analysis of MediaPipe-based measurement system for some human rehabilitation motions. Measurement.

[2] Guo W., Craig O., Difato T., Oliverio J., Santoso M., Sonke J., Barmpoutis A. (2022). AI-driven human motion classification and analysis using laban movement system. Lec. Notes Comp. Sci..

[3] Ni S., Al-qaness M.A.A., Hawbani A., Al-Alimi D., Elaziz M.A., Ewees A.A. (2024). A survey on hand gesture recognition based on surface electromyography: Fundamentals, methods, applications, challenges and future trends. Appl. Soft Comput..

[4] Rahman M.M., Uzzaman A., Khatun F., Aktaruzzaman M., Siddique N. (2025). A comparative study of advanced technologies and methods in hand gesture analysis and recognition systems. Expert Syst. Appl..

[5] Atzori M., Cognolato M., Muller H. (2016). Deep learning with convolutional neural networks applied to electromyography data: A resource for the classification of movements for prosthetic hands. Front. Neurorobot..

[6] Chen X., Li Y., Hu R., Zhang X., Chen X. (2020). Hand gesture recognition based on surface electromyography using convolutional neural network with transfer learning method. IEEE J. Biomed. Health Inform..

[7] Hudgins B., Parker P., Scott R.N. (1993). A new strategy for multifunction myoelectriccontrol. IEEE Trans. Biomed. Eng..

[8] Zhang B., Liang H., Wang S., Wang Y., Li Z. (2023). Gesture recognition of sEMG signal based on GASF-LDA feature enhancement and adaptive ABC optimized SVM. Biomed. Signal Process. Control.

[9] Fan J., Wen J., Lai Z. (2023). Myoelectric pattern recognition using Gramian angular field and convolutional neural networks for muscle–computer interface. Sensors.

[10] Wang N.F., Chen Y.L., Zhang X.M. (2013). The recognition of multi-finger prehensile postures using LDA. Biomed. Signal Process. Control.

[11] Liu J., Zhou P. (2013). A novel myoelectric pattern recognition strategy for hand function restoration after incomplete cervical spinal cord injury. IEEE Trans. Neural Syst. Rehabil. Eng..

[12] Oskoei M.A., Hu H. (2008). Support vector machine-based classification scheme for myoelectric control applied to upper limb. IEEE Trans. Biomed. Eng..

[13] Zhang M., Liu S., Li X., Qu L., Zhuang B., Han G. (2024). Improving sEMG-based hand gesture recognition through optimizing parameters and sliding voting classifiers. Eletronics.

[14] Wei W., Wong Y., Dua Y., Hua Y., Kankanhalli M., Geng W. (2019). A multi-stream convolutional neural network for sEMG-based gesture recognition in muscle-computer interface. Pattern Recognit. Lett..

[15] Wei W., Dai Q., Wong Y., Hu Y., Kankanhalli M., Geng W. (2019). Surface-electromyography-based gesture recognition by multi-view deep learning. IEEE Trans. Biomed. Eng..

[16] Wei W., Hong H., Wu X. (2021). A hierarchical view pooling network for multichannel surface electromyography-based gesture recognition. Comput. Intell. Neurosci..

[17] Yang Z., Jiang D., Sun Y., Tao B., Tong X., Jiang G., Xu M., Yun J., Liu Y., Chen B. (2021). Dynamic gesture recognition using surface EMG signals based on multi-stream residual network. Front. Bioeng. Biotechnol..

[18] Wang S., Huang L., Jiang D., Sun Y., Jiang G., Li J., Zou C., Fan H., Xie Y., Xiong H. (2022). Improved multi-stream convolutional block attention module for sEMG-based gesture recognition. Front. Bioeng. Biotechnol..

[19] Jiang B., Wu H., Xia Q., Xiao H., Peng B., Wang L., Zhao Y. (2024). An efficient surface electromyography-based gesture recognition algorithm based on multiscale fusion convolution and channel attention. Sci. Rep..

[20] Jiang B., Wu H., Xia Q., Li G., Xiao H., Zhao Y. (2025). NKDFF-CNN: A convolutional neural network with narrow kernel and dual-view feature fusion for multitype gesture recognition based on sEMG. Digital Signal Process..

[21] Chen Z., Yang J., Xie H. (2021). Surface-electromyography-based gesture recognition using a multistream fusion strategy. IEEE Access.

[22] Xu L., Zhang K., Yang G., Chu J. (2022). Gesture recognition using dual-stream CNN based on fusion of sEMG energy kernel phase portrait and IMU amplitude image. Biomed. Signal Process. Control.

[23] Shin J., Miah A.S.M., Konnai S., Takahashi I., Hirooka K. (2024). Hand gesture recognition using sEMG signals with a multi-stream time-varying feature enhancement approach. Sci. Rep..

[24] Gan Z., Bai Y., Wu P., Xiong B., Zeng N., Zou F., Li J., Guo F., He D. (2025). SGRN: SEMG-based gesture recognition network with multi-dimensional feature extraction and multi-branch information fusion. Expert Syst. Appl..

[25] Atzori M., Gijsberts A., Castellini C., Caputo B., Hager A.G.M., Elsig S., Giatsidis G., Bassetto F., Müller H. (2014). Electromyography data for non-invasive naturally-controlled robotic hand prostheses. Sci. Data.

[26] Englehart K., Hudgins B. (2003). A robust, real-time control scheme for multifunction myoelectric control. IEEE Trans. Biomed. Eng..

[27] Jiang W., Yin Z. (2015). Human activity recognition using wearable sensors by deep convolutional neural networks. Proceedings of the 23rd ACM international conference on Multimedia.

[28] Zhao X., Sun H., Lin B., Zhao H., Niu Y., Zhong X. (2022). Markov transition fields and deep learning-based event-classification and vibration-frequency measurement for φ-OTDR. IEEE Sens. J..

[29] Krizhevsky A.A., Sutskever I., Hinton G.E. (2017). Imagenet classification with deep convolutional neural networks. Adv. Neural Inf. Process. Syst..

[30] Kang J., Zhu X., Shen L., Li M. (2024). Fault diagnosis of a wave energy converter gearbox based on an Adam optimized CNN-LSTM algorithm. Renew. Energy.

[31] Geng W., Du Y., Jin W., Wei W., Hu Y., Li J. (2016). Gesture recognition by instantaneous surface EMG images. Sci. Rep..

[32] Cheng Y., Li G., Yu M., Du J., Yun J., Liu Y., Liu Y., Chen D. (2020). Gesture recognition based on surface electromyography- feature image. Concurr. Comput. Pract. Exp..

[33] Castellini C., Fiorilla A.E., Sandini G. (2009). Multi-subject/daily-life activity EMG-based control of mechanical hands. J. NeuroEng. Rehabil..

